# A Cold

**Published:** 1892-05

**Authors:** J. J. Waller

**Affiliations:** Oliver Springs, Tenn.


### A COLD.

BY J. J. WALLER, M. D., OLIVER SPRINGS, TENN.

In dealing with the above subject we, of course, are aware of the fact that it has never as yet been clearly defined. Most or very nearly all text books consider it merely as a cause of a number of different pathological conditions, or sometimes the *morbus* at hand is considered a phase or manifestation of what is so familiar among us—"taking cold."

Dismissing further speculation along the line as not germane to the object now in view, I wish to call especial attention to the mechanism by which the effects of taking cold are brought about; and as theories and facts make up the bulk of our medical information, probably to theorize on this subject and draw a corollary of facts from the result, would be the proper manner of procedure.

Any portion or the whole of the body exposed to a cold draught for a varied length of time of course suffers from irritation, and immediately wires the ganglion or centre most intimately connected with that region, through the afferent nerves, and makes known the disturbance there. If the irritation is great (which we will assume to be a fact now) and the whole nervous system has to take cognizance of it, the disturbance is appreciated as an insult, and revenge is at once sought by sending out orders to have the secretions and excretions of the skin locked until peace is made. When the glands of the skin surrender their function, the ramparts of the citadel are taken, the skin becomes in a measure dry and chaffy and loses its usual pliancy which is so essential to health. With the periphery thus in a state of blockade, it is not known by the economy at what time some of the more vital internal organs will suffer; so the nervous system trembles with fear, and we have a form of nervousness as a concomitant symptom of cold. The nervous system still trembling with fear and maddened by the insult of irritation, resolves to carry on the secretions and excretions by precipitating a double duty on the internal mucous membranes or serous membranes as the case may be. So when the nervous system orders a mucous or serous membrane on double duty it revolts at the idea of having a vicarious function to perform, and even refuses to carry on its normal function. It is now that we have the dry stage of cold. When the nervous system locked the secretions and excretions it seemed to not realize the fact that it was at the same time locking in some of the venomous products of destructive metamorphosis which, so to speak, in a state of stagnation, undergo a sort of change and become irritating to the brain and nervous system, thus causing the dull lethargic feeling and indifference to mental and physical exertion, and the aching pains in the limbs.

After a while the mad internal membrane yields to its higher authority, the nervous system, and being overburdened by hyper-secretion and hyper-excretion soon ceases to do its work physiologically and passes into a pathological state, and a catarrh is the result. Thus we may have coryza, pharyngitis, laryngitis, bronchitis, enteritis, etc., if a mucous membrane be involved; pleurisy, pericarditis, etc., if it falls on a serous membrane. Other troubles besides diseases of mucous and serous membranes are brought about by cold, but it is not our purpose to go minutely into them now.

In treating a cold, just bear in mind the mechanism by which it was brought about. The nervous system is willing to compromise on most any plan which includes removal of the offending locked-up excretions. Diaphoretics propose to do that, and on their administration and promise the nervous system unlocks the pores of the skin, and equilibrium is restored.

